# Japanese Application of Bioassays for Environmental Management

**DOI:** 10.1100/tsw.2002.125

**Published:** 2002-03-02

**Authors:** Takashi Kusui

**Affiliations:** College of Technology, Toyama Prefectural University, 5180 Kurokawa, Kosugimachi, Toyama, 939-0398, Japan

**Keywords:** bioassay, ecotoxicology, environmental management

## Abstract

The increasing number of existing and new chemicals demands ecotoxicological data as well as toxicological data for pre- and postmarketing risk assessments. Although human health has been the major concern in Japanese environmental management, ecosystem health is becoming the big issue as the need for preserving the diversity of ecosystems has been recognized. This recognition is changing the regulatory framework in Japan, resulting in new actions toward establishment of water-quality standards for aquatic organisms and ecotoxicological assessment of existing chemicals. At the same time, the need to assess complex liquids that contain several kinds of chemicals is increasing. The ecotoxicological study of Japanese effluents shows that the present chemical-specific standards are not enough to protect aquatic ecosystems. These two factors encourage the application of ecotoxicological tests as well as the toxicological data.

## INTRODUCTION

With an increasing number of chemicals in use, the risks associated with these chemicals have been potentially significant to human and ecosystem health. According to the Chemical Abstract Service, about 24 million chemicals had been registered as of May 2000, and about 1.3 million new chemicals have been registered every year for the last 5 years. It is estimated that about 60,000 chemicals have been produced and are used in our daily lives. The survey conducted by the Japanese Environmental Agency showed that about 40% of targeted chemicals (287/752) have been detected in the environment (i.e., water, sediment, and organisms), suggesting that more than 10,000 chemicals might remain in the environment.

To reduce the risks associated with these chemicals, there are several strategies. Premarketing control and environmental quality standards have been major tools so far; however, only human health has been the major concern in Japan. In some cases, the ecosystem is more susceptible to chemicals than humans. Recently the diversity of ecosystems has been recognized, and ecosystem health has been regarded as being as important as human health. The objective of this paper is to give a brief overview of the application of the bioassays and their data for environmental management in Japan, especially in ecotoxicological aspects.

## REGULATORY FRAMEWORK FOR ECOLOGICAL RISK

Until recently, premarketing regulations, such as the Chemical Substances Control Law, the Agricultural Chemicals Regulation, and the Ocean Pollution Prevention Law, have been the only regulatory systems that deal with ecotoxicological assays. The Chemical Substances Control Law aims to control the manufacture, import, and use of new chemicals that are not easily biodegradable, easily accumulated in the body, and may pose a threat to human health; however fish are used only to examine the accumulation properties of chemicals, not to assess the chemicals' toxicity to fish. The Agricultural Chemicals Regulation aims to prevent the damage to aquatic animals and plants caused by agricultural chemicals. The withholding standards for the criteria have been established based on fish acute toxicity (48h-TLm). However, even registered agricultural chemicals are divided into three levels on the basis of their TLm values, only the precaution for use is determined based on the classification level. The Ocean Pollution Prevention Law provides the criteria for oil dispersant used for oil spills. The toxicity to algae and fish are considered.

Environmental quality standards and other standards (i.e., effluent, waste, and soil) work as postmarketing regulations. Although environmental quality standards are derived from the latest knowledge on toxicity and modern risk-assessment methodologies, these standards focus only on human health.

## NEW MOVEMENT TOWARD ECOTOXICOLOGICAL RISK MANAGEMENT

After the enactment of the Basic Environmental Law in 1993, a new movement toward the preservation of aquatic organisms has occurred in regulatory agencies.

The first example is the Law Concerning the Reporting of the Release into the Environment of Specific Chemical Substances and Promoting Improvements in Their Management (1999), or the so-called PRTR system. The purpose of the law is to create a framework for businesses to improve and strengthen the management of chemical substances known to be harmful — including those for which a causal effect detrimental to the human body has not been established — and to prevent environmental pollution. The important aspect of this new law is that the criteria for selecting chemicals include the level of toxicity to aquatic organisms such as algae, daphnia, and fish.

The second movement is the hazard assessment of existing chemicals that might be harmful to aquatic organisms. The working group on the ecological risk assessment of chemicals was established in 1997 at the request of the Ministry of Environment (formally the Environmental Agency). Chemicals have been selected according to production, ecotoxicity, and the possibility of presence in the environment, and the predicted no-effect concentration has been calculated for each chemical (Fig. 1). These PNECs, combined with the predicted environmental concentrations, have been used for ecotoxicological risk assessment.

The third action is to establish environmental quality standards for aquatic ecosystem protection. The interim report released in December 2000 recommended that three types of protected areas should be designated, such as protected areas for *Salvelinus* and *Oncorhynchus, Cyprinus* and *Carassius*, and marine species. The environmental quality standards will be established for protected species[1]. Since 2001, the environmental quality standards for 81 chemicals regarded as high risk for aquatic organisms have been calculated.

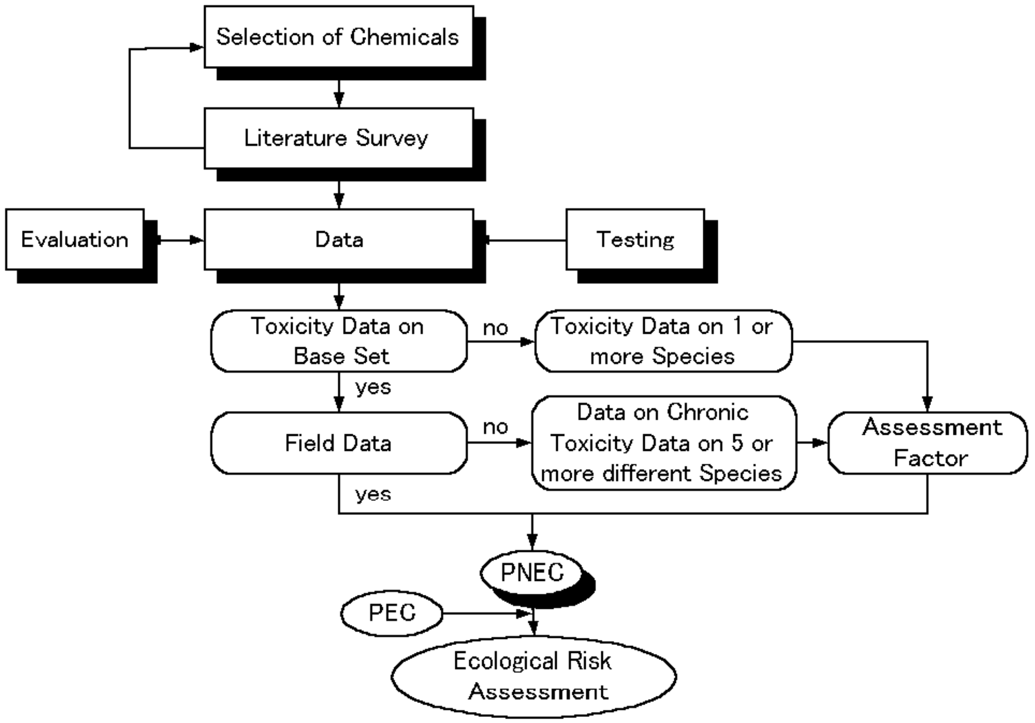

**FIGURE 1.** Ecological risk assessment flow.

Methods outlined in OECD chemical test guidelines are used extensively in Japan to conduct ecotoxicological testing. On top of that, guidelines for ecotoxicological testing by the Environmental Agency, the Japanese Industrial Standard Testing Method for Drinking Water and the Testing Method for Sewage contain a variety of ecotoxicological testing methodologies. They are used on an arbitrary basis because there is no regulatory standard for known toxicity at present. However, the use of toxicity testing is increasing because of the increased need to assess complex media such as effluent, drinking water, or leachates from landfill sites that may contain various known and unknown chemicals. The Japanese Society of Environmental Toxicology, an academic organization launched in 1997, has approximately 400 members who are contributing to the research, development, and application of ecotoxicological testing.

As outlined above, ecotoxicological risk is becoming a big issue in Japan. This trend will change the environmental policy dramatically within several years.

## ECOTOXICOLOGICAL ASSESSMENT OF EFFLUENT

Pre- and postmarketing controls mentioned above deal only with specific chemicals individually; however, complex liquid media such as effluents and leachates contain several kinds of chemicals, and it is difficult to assess the hazards of these media from chemical concentrations. In these cases, comprehensive tools such as bioassays are useful to give integrated information about the effects (Fig. 2)[2]. In the last decade, there have been many discussions about the use of bioassays for environmental management. To control the industrial discharge, North American and European countries have regulatory standards based on biological testing as well as chemical concentrations. Although many studies have adapted the ecotoxicological approach to assess industrial discharges in Japan, it takes some time to get a general acceptance of the bioassay approach in environmental management from the regulatory, industrial, and academic arenas.

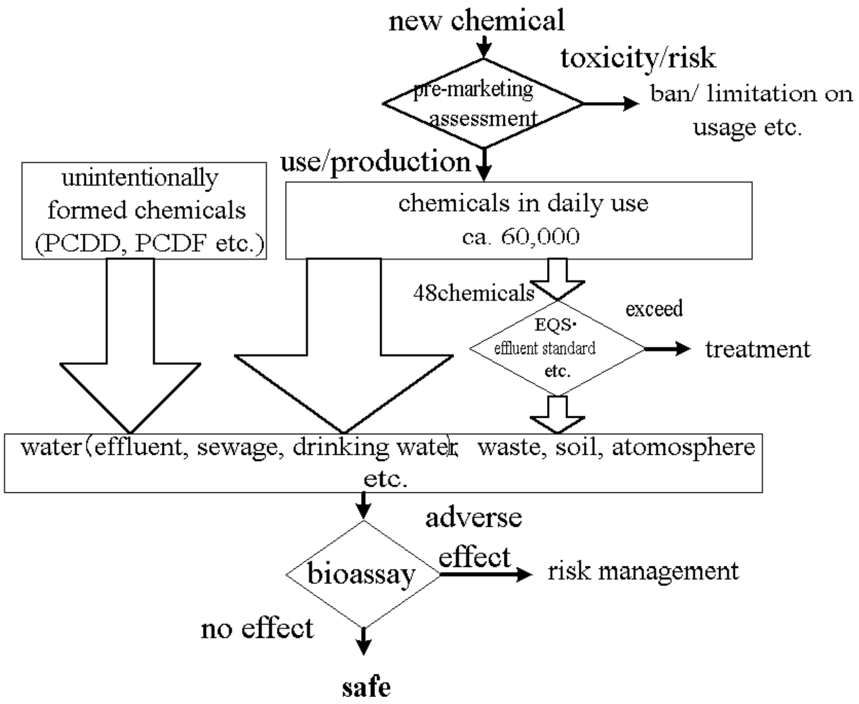

**FIGURE 2**. Role of bioassays in environmental management.

Some preliminary studies, however, show the importance of bioassays as management tools. One example is the ecotoxicological assessment of 18 industrial effluents and 2 sewage effluents in Toyama Prefecture, Japan, that employed five small-scale ecotoxicological bioassays[3]. Algal assays and hydra acute sublethal assays had the most sensitive detection capacity and showed positive results in 80 and 60% of effluents tested, respectively. The comparison of six metal bioassay results and measured concentrations in effluents suggested the significant contribution of two metals ($Zn^{2+}$ and $Cu^{2+}$) to the toxicity of effluent from a surface processing factory. By integrating the results of five bioassays in each effluent, the relative ecotoxicological potencies were compared. Finally, the comparison of toxic loading (potency × flow) showed the relatively high contributions from several large dischargers into the aquatic environment. Based on the Potential Ecotoxic Effects Probe index values[4], Japanese effluents show lower values (ranging from 0 to 5.2) than those of Canadian effluents (ranging from 0 to 7.2) (Fig. 3). Another example is the report on the ecotoxicological assessment of Japanese pulp and paper mill effluents[5]. The important finding of these studies is that some of these effluents showed significant ecotoxicological effects even though they meet the present regulatory effluent standards.

## CONCLUSION

The increasing number of existing and new chemicals demands ecotoxicological data as well as toxicological data for pre- and postmarketing risk assessment. Although human health has been the major concern in Japanese environmental management, ecosystem health is becoming a big issue as the need to preserve the diversity of ecosystems has been recognized. This recognition is reflected in the changing regulatory framework in Japan. At the same time, the need to assess complex liquid media that contain several chemicals is increasing. These two factors demand the application of ecotoxicological tests as well as their data.

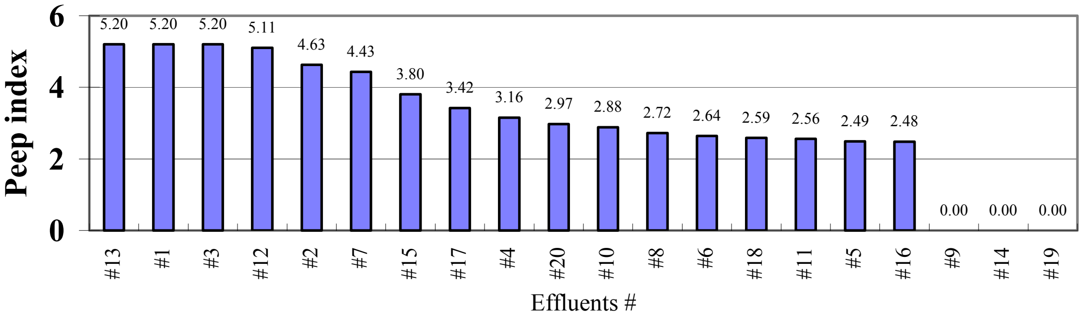

**FIGURE 3.** PEEP (Potential Ecotoxic Effects Probe) values of 20 Japanese effluents. #1: pulp and paper, #2: chemical production, #3: pharmaceuticals, #4: surface processing, #5: pharmaceuticals, #6: recycled paper, #7: metal processing, #8: meat processing, #9: sewage treatment, #10: textile, #11: recycled pulp, #12: chemical production, #13: aluminum, #14: semiconductor, #15: chemicals production, #16: aluminum, #17: recycled paper, #18: refinery, #19: laundry, #20: sewage treatment.
